# Electrochemical Cation-Swing for Carbon Capture under Ambient Conditions

**DOI:** 10.1021/acscentsci.3c01064

**Published:** 2023-09-06

**Authors:** Yayuan Liu

**Affiliations:** Department of Chemical and Biomolecular Engineering, Johns Hopkins University, Baltimore, Maryland 21218, United States

Mitigating anthropogenic CO_2_ emissions to combat climate change necessitates carbon capture from both industrial point emitters and the ambient environment. The incumbent carbon capture technology is amine scrubbing, which relies on energy-intensive thermal swings to uptake and then release CO_2_ using aqueous amine solvents. In this issue of *ACS Central Science*, Gallant and co-workers advance a new strategy to reversibly modulate the CO_2_ loading on nonaqueous amine solvents at room temperature driven solely by electricity.^1^

**Figure 1:**
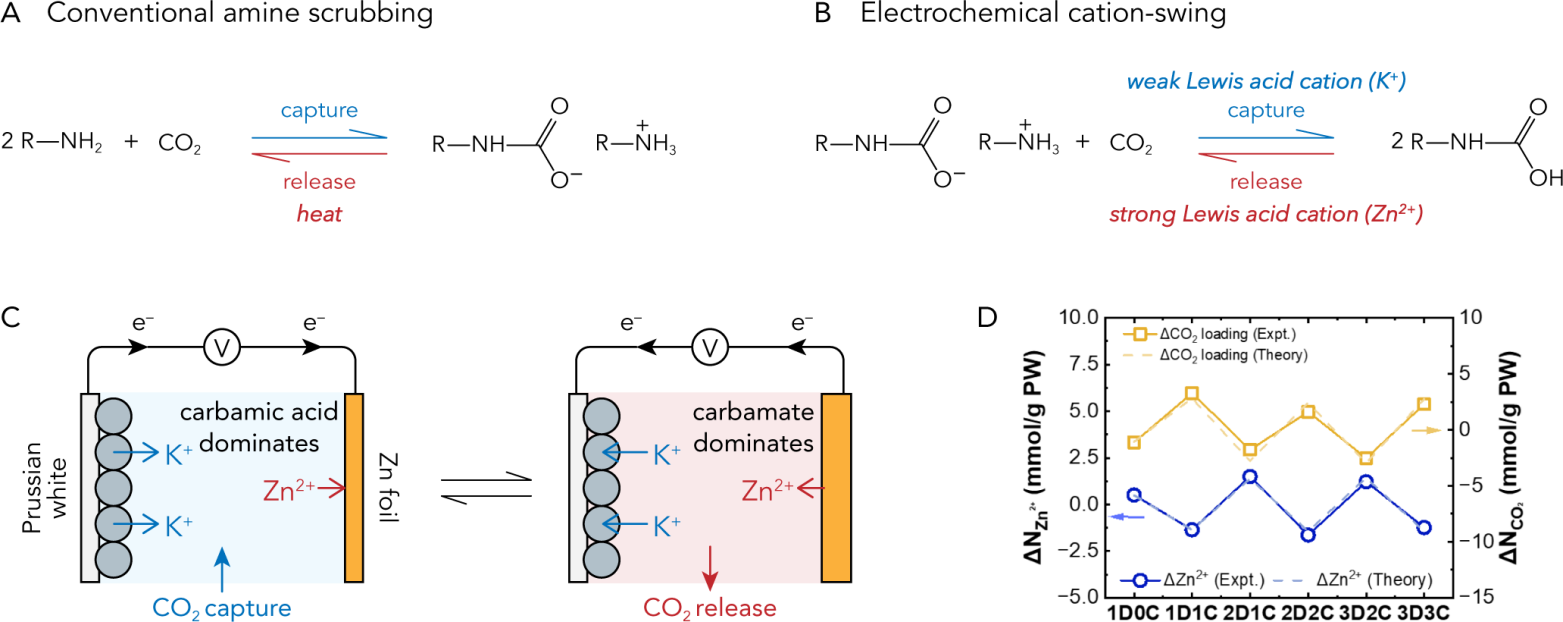
(A) Reaction scheme of conventional thermochemical amine scrubbing. (B) The new electrochemical cation-swing process for reversible CO_2_ capture and release using nonaqueous amine solvents proposed by Gallant and co-workers. (C) Prussian white and Zn foil were selected as electrodes to demonstrate the proposed cation-swing process. (D) Changes of Zn^2+^ concentration and CO_2_ loading on amines during electrochemical cycling. Panel D was reproduced with permission from ref (1). Copyright 2023 The Authors. Published by American Chemical Society.

Thermochemical amine scrubbing, a largely matured technology, captures CO_2_ from dilute sources in the form of carbamates at ∼40 °C and releases a high-purity CO_2_ outlet stream for storage or utilization at ∼120 °C (Figure 1A).^2^ Despite the broad consensus on the importance of carbon capture, amine scrubbing has not been implemented fast enough to the scale necessary to close the anthropogenic carbon cycle.^3^ One major technical barrier is the high energy excursions associated with thermal amine regeneration, motivating the search for new carbon capture mechanisms driven by nonthermal stimuli. Over the past decade, with the accelerated deployment of utility-scale wind and solar power and the consequent plunging cost of renewable electricity, electrochemically mediated carbon capture methods have galvanized growing research interests. The reversible capture and release of CO_2_ induced by electrochemical driving forces offer distinct advantages, such as ambient operating conditions to minimize energy cost and modularity to accommodate the multiscale needs for carbon capture.^4^ Various mechanisms have been previously reported for electrochemically mediated carbon capture.^4^ Nevertheless, as an emerging research field, further explorations of new approaches remain crucial.

The Gallant group at the Massachusetts Institute of Technology pioneers the study of amine speciation in nonaqueous environments. In prior work,^5^ they observed that the reaction between amine and CO_2_ in neat dimethyl sulfoxide (DMSO) solvent favors the formation of carbamic acid (1 mol CO_2_/mol amine). However, the addition of supporting salts can induce substantial transformation of carbamic acid into ionic ammonium carbamate (0.5 mol CO_2_/mol amine), and the extent of such a respeciation process is dictated by the Lewis acidity of the supporting salt cation. This speciation change implies the feasibility of releasing one CO_2_ from every two amine molecules if carbamic acid can be fully converted to carbamates. Correspondingly, in their new carbon capture scheme (Figure 1B), a pair of electrodes alternates the identity of cations in nonaqueous amine solution between strong and weak Lewis acids to toggle amine species reversibly between carbamic acid and carbamate. To fundamentally interrogate factors influencing this cation-swing process, detailed nuclear magnetic resonance spectroscopy (NMR) studies were conducted to screen candidate cations (Li^+^, K^+^, Ca^2+^, Mg^2+^, Zn^2+^), and the thermodynamic driving force of the carbamic acid-to-carbamate conversion was investigated via gas-flow reaction microcalorimetry. Through the exploration, K^+^ and Zn^2+^ were identified as candidate weak and strong Lewis acid cations, respectively. Gas sampling experiments showed that the CO_2_ loading change between a 100% K^+^ or a 100% Zn^2+^ ethoxyethylamine (EEA)/DMSO solution approaches ∼40%, closest to the theoretical maximum of 50%.

This new electrochemical cation-swing mechanism lays the foundation for a potential drop-in replacement for existing amine scrubbing, which can exploit the excellent removal efficiencies of conventional amine scrubbers while reducing parasitic energy and capital costs. To demonstrate a proof-of-concept CO_2_ capture and release process, the authors selected K^+^-intercalation Prussian white and metallic Zn as the electrode pair and EEA/DMSO electrolyte containing dual KTFSI/Zn(TFSI)_2_ salt as the carbon capture solvent (Figure 1C). The demonstrated batch process was carried out inside an electrochemical cell with pure CO_2_ headspace. By sampling the electrolyte at different charge/discharge states, the authors indeed observed a direct correlation between Zn^2+^ concentration and CO_2_ loading, which agrees well with the theoretical values (Figure 1D). The electrical energy consumption of the cation-swing process was calculated to be ∼22–39 kJ/mol CO_2_, although the current densities remain low for practical applications (0.1–0.5 mA cm^–2^). Besides, the Prussian white–Zn foil electrode pair exhibited high Coulombic efficiency and capacity retention during repeated cycling in the EEA/DMSO electrolyte, indicating the good reversibility of the system.

This work undoubtedly opens up abundant opportunities for fundamental research and engineering optimization. As the authors pointed out, the throughput of the process hinges on the capacity of the electrodes; finding high-capacity intercalation materials that can operate reversibly at high currents will be one of the prerequisites for the practical implementation of this concept. Moreover, given the inexhaustive combination of amines, nonaqueous solvents, and Lewis acid cations, it is important to optimize the electrolyte formulation taking into account not only the extent of CO_2_ loading modulation but also other key metrics such as carbamate solubility, viscosity, etc. With more optimized electrodes and electrolytes, it would be interesting to demonstrate a flow-based electrochemical carbon capture prototype, which will likely be the configuration required for real applications.^6^ Finally, future work needs to interrogate the effect of water and other common gas stream impurities on the cation-swing process and probe the failure mechanisms in detail to enable informed mitigation strategies. Given the seriousness of the climate change problems, explorations on innovative approaches to carbon capture are critically needed, and scientists and engineers across different fields need to work together to assess the potential of new ideas and translate promising ones into practical technologies.
